# 3D porous polymers for selective removal of CO_2_ and H_2_ storage: experimental and computational studies

**DOI:** 10.3389/fchem.2023.1265324

**Published:** 2023-09-07

**Authors:** Muath S. Al-Bukhari, Ismail Abdulazeez, Mahmoud M. Abdelnaby, Isam H. Aljundi, Othman Charles S. Al Hamouz

**Affiliations:** ^1^ Chemistry Department, King Fahd University of Petroleum and Minerals, Dhahran, Saudi Arabia; ^2^ Interdisciplinary Research Center for Membranes and Water Security, King Fahd University of Petroleum and Minerals, Dhahran, Saudi Arabia; ^3^ Interdisciplinary Research Center for Hydrogen and Energy Storage, King Fahd University of Petroleum and Minerals, Dhahran, Saudi Arabia; ^4^ Chemical Engineering Department, King Fahd University of Petroleum and Minerals, Dhahran, Saudi Arabia

**Keywords:** 3D porous polymers, global warming, flue gas purification, CO_2_ capture, H_2_ storage

## Abstract

In this article, newly designed 3D porous polymers with tuned porosity were synthesized by the polycondensation of tetrakis (4-aminophenyl) methane with pyrrole to form **M1** polymer and with phenazine to form **M2** polymer. The polymerization reaction used *p*-formaldehyde as a linker and nitric acid as a catalyst. The newly designed 3D porous polymers showed permanent porosity with a BET surface area of 575 m^2^/g for **M1** and 389 m^2^/g for **M2**. The structure and thermal stability were investigated by solid ^13^C-NMR spectroscopy, Fourier-transform infrared (FT-IR) spectroscopy, and thermogravimetric analysis (TGA). The performance of the synthesized polymers toward CO_2_ and H_2_ was evaluated, demonstrating adsorption capacities of 1.85 mmol/g and 2.10 mmol/g for CO_2_ by **M1** and **M2**, respectively. The importance of the synthesized polymers lies in their selectivity for CO_2_ capture, with CO_2_/N_2_ selectivity of 43 and 51 for **M1** and **M2**, respectively. **M1** and **M2** polymers showed their capability for hydrogen storage with a capacity of 66 cm^3^/g (0.6 wt%) and 87 cm^3^/g (0.8 wt%), respectively, at 1 bar and 77 K. Molecular dynamics (MD) simulations using the grand canonical Monte Carlo (GCMC) method revealed the presence of considerable microporosity on **M2**, making it highly selective to CO_2_. The exceptional removal capabilities, combined with the high thermal stability and microporosity, enable **M2** to be a potential material for flue gas purification and hydrogen storage.

## Graphical Abstract

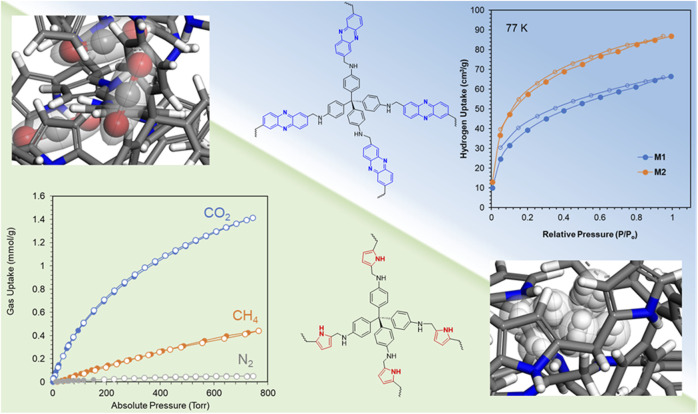

## 1 Introduction

Global warming caused by the elevated levels of CO_2_ has garnered significant attention in recent years. The elevated levels of CO_2_ have become a serious problem due to their hazardous effects on the environment; these effects encompass a gradual increase in the temperature of the earth, resulting in droughts, fluctuations in the weather, and elevated oceanic water levels and ocean acidification (Feldman et al., 2015; Leal et al., 2018; Zakeri et al., 2022). During the last 40 years, the concentration of CO_2_ has increased tremendously from 319 ppm to 414 ppm in 2021, setting a new record, and is estimated to increase to 800 ppm within the next 100 years if we continue relying on fossil fuels as a primary energy source (Mercer, 1978; Feldman et al., 2015; Abdelhakim et al., 2022). Fossil fuels, serving as energy sources, are typically divided into three types: natural gas, coal, and petroleum. Upon combustion, they release CO_2_, SOx, and NOx gases; mercury; and various particulates that cause pollution in the environment and have a large impact on human health (Khraisheh et al., 2020; Perera and Nadeau, 2022). Due to the major concern for the environment and human health, several methods and techniques have been identified to reduce the effect of CO_2_ (Taylor et al., 2020; Long et al., 2021). These techniques and methods include finding new sources of energy, such as hydrogen gas, as an alternative energy source, reducing energy consumption by increasing energy efficiency, and finding new methods for capturing CO_2_ (Kar et al., 2022; Paramati et al., 2022). Capturing CO_2_ is one of these methods and has garnered considerable attention over the years. Two major methods have been used: chemisorption of CO_2_, which involves the formation of a chemical bond between CO_2_ and the adsorbent. Such an example for chemisorption is the absorption of CO_2_ by liquid amines which is the most commonly used method by refineries to capture CO_2_ from natural gas streams, their operation is non-costeffective and requires high energy for regeneration. Furthermore, degradation of the liquid amines thermally and oxidatively causes corrosion in refinery setups (Bobek et al., 2016; Dey et al., 2017; Kong et al., 2019). The other major technique that is emerging is capturing CO_2_ by physisorption. Physisorption is a process where CO_2_ is bonded weakly with the adsorbent by weak van der Waals forces of attraction, which allows the sorbent to be capable of reversibly adsorbing CO_2_ from flue gas streams by solid sorbents (Plaza et al., 2007; Oschatz and Antonietti, 2018; Kong et al., 2019). Solid sorbents have been developed through the years, and several key features should be included in the design of these sorbents for efficient CO_2_ capture, such as i) high sorption capacity, ii) selectivity, and iii) adequate stability in the presence of contaminants (Zou et al., 2017; Abdelnaby et al., 2019; Khraisheh et al., 2020). Different classes of solid sorbents have emerged as promising materials for CO_2_ reduction, such as metal–organic frameworks (MOFs), covalent organic frameworks (COFs), zeolites, carbonaceous materials, such as activated carbon, and porous organic polymers (POPs) (Cheung and Hedin, 2014; Gadipelli and Guo, 2015; Lohse and Bein, 2018; Zhao et al., 2018; Qasem et al., 2020). POPs are an interesting class of materials that possess excellent features such as low density, high surface area with a tunable pore size distribution, good thermal and chemical stability, and synthetic versatility (Zou et al., 2017; Gao et al., 2019; Gu et al., 2022). These features are considered requirements for the selective removal of CO_2_ from flue gas and natural gas streams (Rufford et al., 2012; Ahmed et al., 2015; Alloush et al., 2022). In our endeavor to design and synthesize porous organic polymers for CO_2_ capture and hydrogen storage, we demonstrate the design and synthesis of new 3D porous organic polymers with tuned porosity in this study. The synthesized 3D polymers were evaluated for their CO_2_ and H_2_ adsorption capabilities and for their selectivity of CO_2_ over N_2_ and CH_4_ to assess their potential use in flue gas and natural gas treatment.

## 2 Experimental

### 2.1 Materials and methods

Tetrakis (4-aminophenyl) methane (99%), phenazine (99%), and pyrrole (98%) were all purchased from Sigma-Aldrich Co. *p*-Formaldehyde (PF, ≥99.9% purity) was purchased from Fluka™ AG. Nitric acid (65%wt.) and N,N-dimethylformamide (DMF, 99% purity) were obtained from Alpha Chemika™. Methanol (MeOH, ≥99.9% purity) was acquired from Merck Millipore™. Except for pyrrole, which was distilled at 150°C immediately before use, all chemicals were used as received. Ultrahigh-purity-grade nitrogen (N_2_, 99.999%), helium (He, 99.999%), and high-purity carbon dioxide (CO_2_, 99.9%) gases were supplied by Abdullah Hashem Industrial Co., Saudi Arabia. Natural abundance solid-state ^13^C-NMR spectra were collected using a Bruker 400 MHz spectrometer set to 125.65 MHz at room temperature (11.74 T). Samples were packed into 4 mm zirconium oxide rotors. Cross-polarization and high-power decoupling were used. The pulse delay was 2.5 s, and the magic angle spinning rate was 10 kHz. A PerkinElmer FT-IR spectrometer was used to obtain FT-IR spectra. FT-IR spectra were obtained in the range of 4,000–400 cm^−1^ using a PerkinElmer 16F PC FT-IR spectrometer and solid potassium bromide (KBr) pellets (mid-IR region). TGA was performed using the STA 429® (NETZSCH group, Germany) thermal analyzer. All gas uptake measurements were performed on the Quantachrome® Autosorb IQ instrument, and isotherms were obtained at 273 K and 298 K.

### 2.2 Synthesis

In a typical experiment (Abdelnaby et al., 2018), tetrakis (4-aminophenyl) methane (2.73 × 10^−3^ mol, 1.0 g) and pyrrole (0.0109 mol, 0.73 g) were stirred in a 50-mL round-bottomed flask equipped with a magnetic bar containing 25 mL DMF until a homogeneous solution was obtained. *p*-Formaldehyde (0.02187 mol, 0.66 g) and nitric acid (10% of p-formaldehyde; 0.002187 mol, 0.199 g) were then added to the reaction mixture. The reaction mixture was flushed with N_2_ gas and sealed and stirred for 24 h at 90°C. Once the reaction was completed, the product was filtered and washed with methanol for 3 days with continuous exchange of methanol to ensure the removal of any monomers or unreacted materials left in the reaction. The product was vacuum-dried at 90°C for 24 h to get **M1** as a fine black powder (yield % = 65%). **M2** was obtained as a bright yellow powder (yield % = 49%) under similar reaction conditions with tetrakis (4-aminophenyl) methane, phenazine (**M2**), and *p-*formaldehyde taken at a molar ratio of 1:4:8 and 10 mol% of nitric acid relative to *p*-formaldehyde. The yield of the polymerization reaction was calculated as the mass of the product relative to the mass of all reactants.

### 2.3 MD simulation procedure

Molecular dynamics simulations (Supplementary Material) were performed to reveal the underlying mechanism of adsorption of CO_2_, CH_4_, and N_2_ gases by the polymers **M1** and **M2**. The structural geometries of the polymers were built and optimized using the smart algorithm in the Forcite module of Materials Studio 8.0 software. The COMPASS II force field (Sun et al., 2016) was adopted, while the self-consistent field (SCF) convergence threshold, maximum force tolerance, and energy tolerance were set to 1.0 × 10^−5^ Ha, 0.001 Ha/Å, and 1.0 × 10^−5^ Ha, respectively. Thereafter, using the “Locate” task bar on the Sorption module, the suitable adsorption sites of the gases on **M1** and **M2** were identified, and the adsorption capacities were estimated based on the principle of simulated annealing using the GCMC method (Aljamaan et al., 2017; Song et al., 2018). The adsorption isotherms at 273.15, 298.15, and 313.15 K were calculated using the Langmuir fitting equation:

$$y=\frac{abx}{1+bx},$$

where *a* is the limit of adsorption capacity in mmol/g and *b* is the adsorption constant in MPa^−1^. The estimated adsorption capacities were given in the units of average molecules/cell and were converted to the amount of gas adsorbed in mmol/g using the following equation (Zhang et al., 2021):

$$Amount\;adsorbed\;\left(mmol/g\right)=\frac{loading\;molecules}{{Mw}_{cell}\left(g/mol\right)}\times 1000,$$

where Mw_cell_ is the relative molecular mass of **M1** and **M2** polymers in the constructed supercell.

## 3 Results and discussion

### 3.1 Synthesis and characterization

**SCHEME 1:**
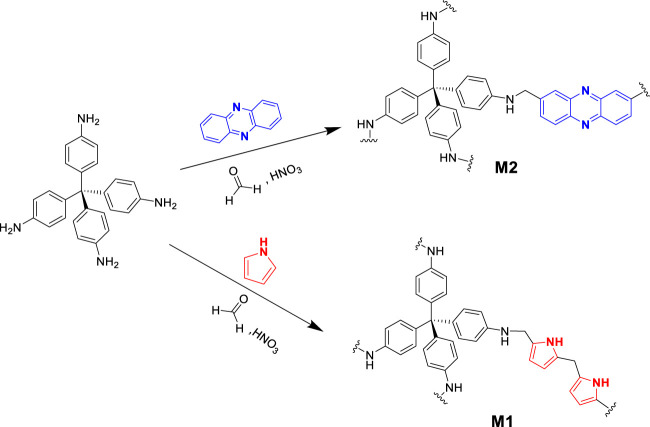
Synthesis scheme for 3D porous polymers.

This paper describes two new 3D porous amine-based polymers. The polymerization method was based on a modified Mannich polycondensation reaction, with tetrakis (4-aminophenyl) methane added as a common component in the polymers. The polymers were realized by polymerizing tetrakis (4-aminophenyl) methane with pyrrole to obtain **M1** and phenazine to form **M2**. The polymerization reaction was conducted using DMF as a solvent and concentrated HNO_3_ as a catalyst (Scheme 1).

**FIGURE 1:**
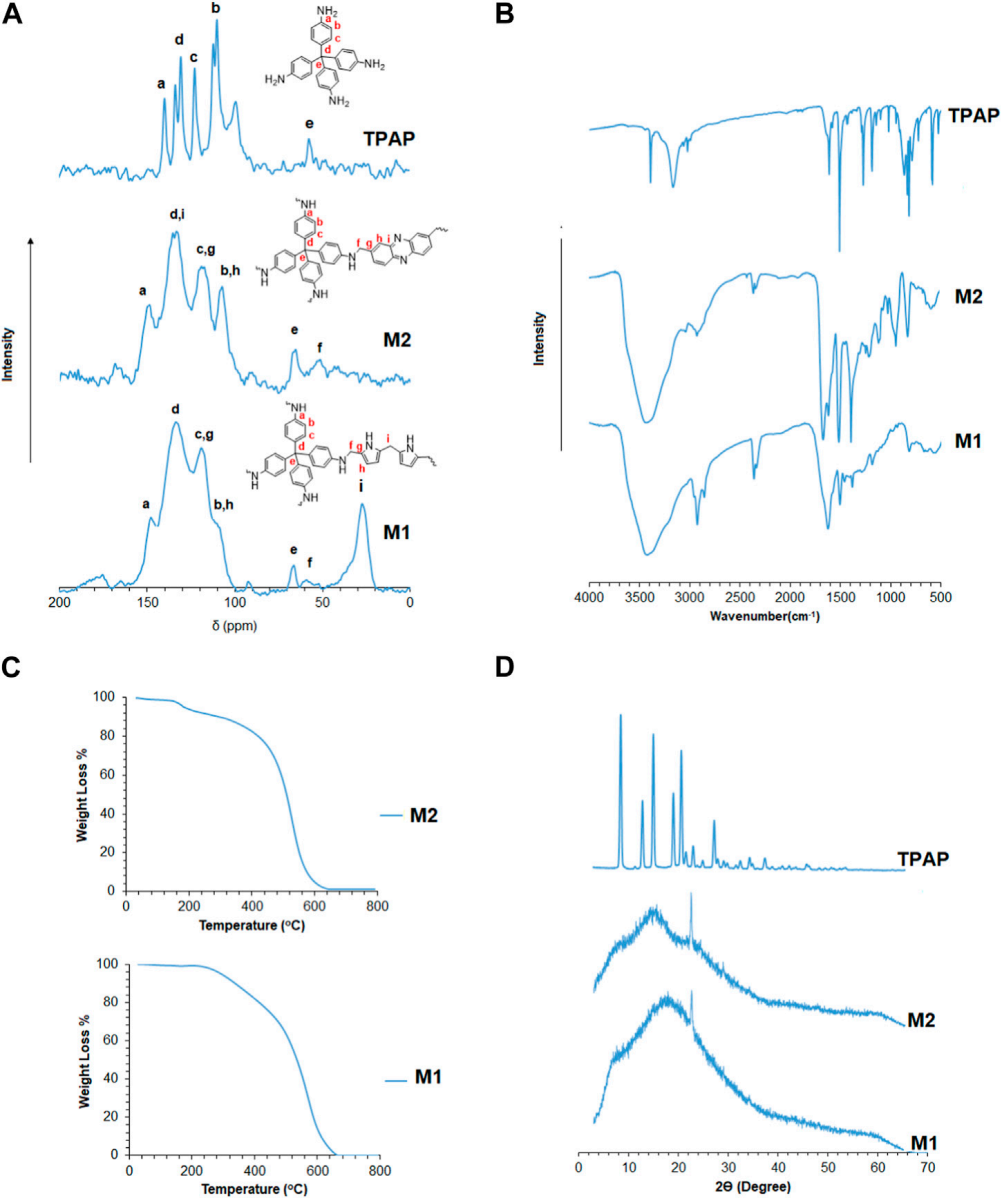
**(A)** Solid ^13^C-NMR CP/MAS spectra for 3D porous polymers. **(B)** FT-IR spectra for 3D porous polymers and identification of peaks. **(C)** Thermogravimetric analysis of 3D porous polymers. **(D)** Powder X-ray diffraction of 3D porous polymers.

The structural features of the polymers were characterized by solid ^13^C-NMR, as shown in Figure 1A. The peaks residing from 100 ppm to 150 ppm correspond to the aromatic carbons of pyrrole, phenazine, and tetrakis (4-aminophenyl) methane. A peak at 65 ppm corresponds to the quaternary carbon of tetrakis (4-aminophenyl) methane, linking the four aniline moieties. A peak at 55 ppm corresponds to the methylene linkage (-CH_2_-) between tetrakis (4-aminophenyl) methane and pyrrole or phenazine. A peak at ∼30 ppm corresponds to the methylene linkage (-CH_2_-) present between pyrrole and pyrrole moieties (Luo et al., 2012; Abdelnaby et al., 2018). Figure 1B represents the FT-IR spectra for the synthesized polymers. The figure shows a broadband in the region from 3,300 to 3,500 cm^−1^ resulting from the overlap between both the 1° amine (-NH_2_) stretching vibrations of the aniline moiety in the tetrakis (4-aminophenyl) methane monomer and the 2° amine (-NH-) stretching band of the pyrrole moiety. The bands between 1,400 and 1,700 cm^−1^ correspond to the aromatic -C=C- and -C=N- stretching vibrations of phenazine, pyrrole, and aniline moieties. A band at 1,631 cm^−1^ attributed to -NH_2_ scissoring can be observed overlapping with the -C=C- aromatic vibrational bands that appear in the same region. Both -NH_2_ and -NH- wagging bands are at 694 and 755 cm^−1^, respectively (Wu et al., 2002; Tian et al., 2009). Figure 1C shows the powder X-ray diffraction patterns of the 3D porous polymers. The powder X-ray diffraction patterns reveal the amorphous nature of the synthesized polymers with a broad signal at ∼15° 2Ө with some degree of crystallinity shown by the signal at ∼ 22° 2Ө present in **M1** and **M2** (Wei et al., 1992; Errahali et al., 2014). Figure 1D reveals the good thermal stability of the synthesized polymers, which could be related to the stiff cross-linked structures of **M1** and **M2**. The thermograms in Figure 4D show a 5% weight loss of small, trapped molecules in **M2** up to 200°C, followed by a second degradation at ∼500°C, where the degradation of the polymer structure occurs by the loss of the methylene linkages, followed by the degradation of the polymer backbone. On the other hand, **M1** begins to thermally degrade at ∼300°C up to 440°C, which may be attributed to the loss of the methylene linkages between the moieties, followed by the complete degradation of the polymer structure at ∼600°C (Li et al., 2014; Yan et al., 2016).

### 3.2 Porosity of the 3D porous polymers

**FIGURE 2:**
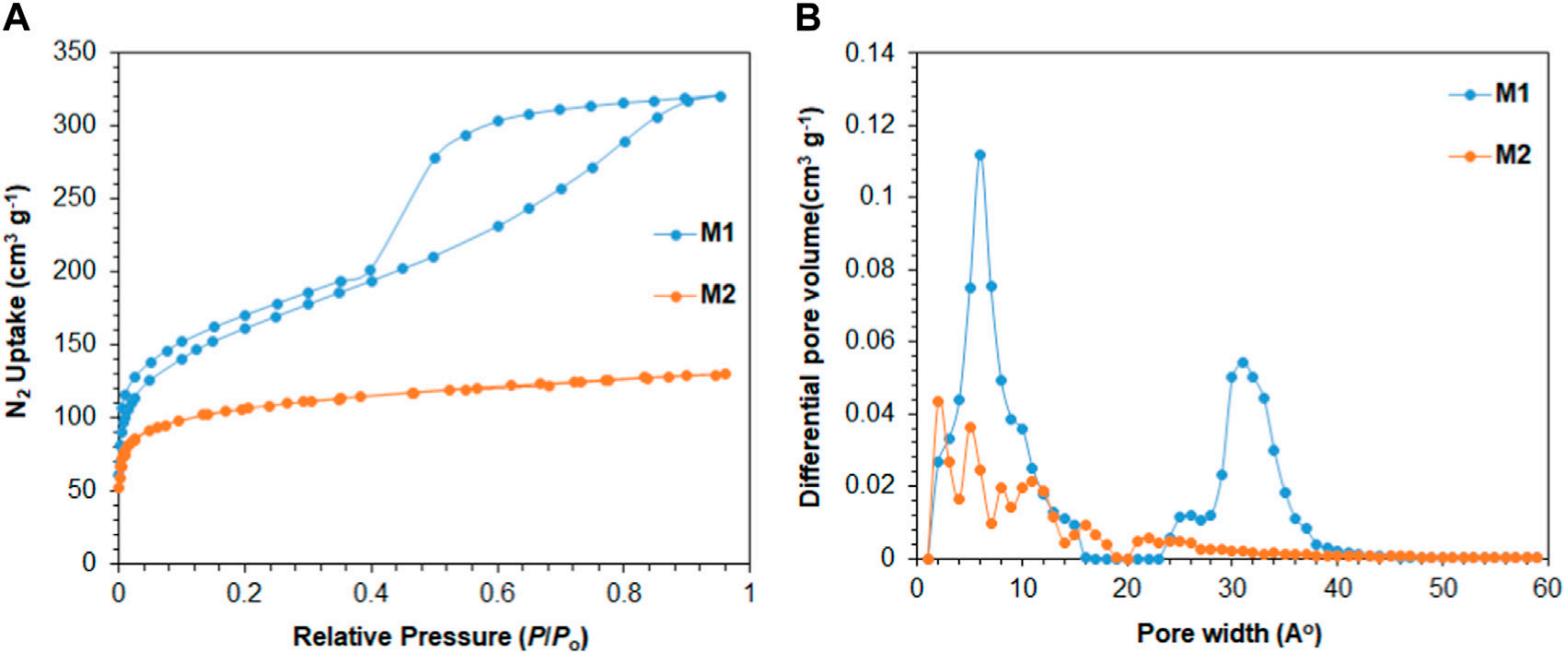
**(A)** Nitrogen adsorption/desorption isotherms of 3D porous polymers at 77 K; **(B)** pore size distribution using DFT.

As shown in Figure 2A, the nitrogen adsorption/desorption isotherms suggest that **M1** and **M2** are porous in nature. **M1** polymer shows permanent porosity with a BET surface area of 575 m^2^/g. The BET isotherm of **M1** suggests that the polymer exhibits Type I characteristics with a steep increase in nitrogen uptake at low relative pressure (*P*/*P*_*0*_ < 0.05). The hysteresis in the **M1** isotherm suggests the high interaction between M1 porous polymer and N_2_ molecules, which could be due to the entrapment of N_2_ molecules in the pores of M1 that leads to the hysteresis found in the adsorption/desorption isotherm (Li et al., 2022). The pore size distribution analysis based on density functional theory (DFT) calculations reveals two distinct regions in the **M2** porous polymer. As shown in Figure 2B, there is a prominent peak at approximately 10 Å, indicating the presence of micropores, and another strong peak at an average pore width of approximately 33 Å, representing the mesoporous region. **M2** polymer shows a permanent porosity with a BET surface area of 389 m^2^/g. The nitrogen adsorption isotherm of **M2** suggests that the polymer is microporous in nature and exhibits Type I characteristics. Further examination using DFT calculations reveals that the apertures of **M2** polymer are mainly in the range of micropores with pore widths less than 20 Å, as shown in Figure 2B.

**FIGURE 3:**
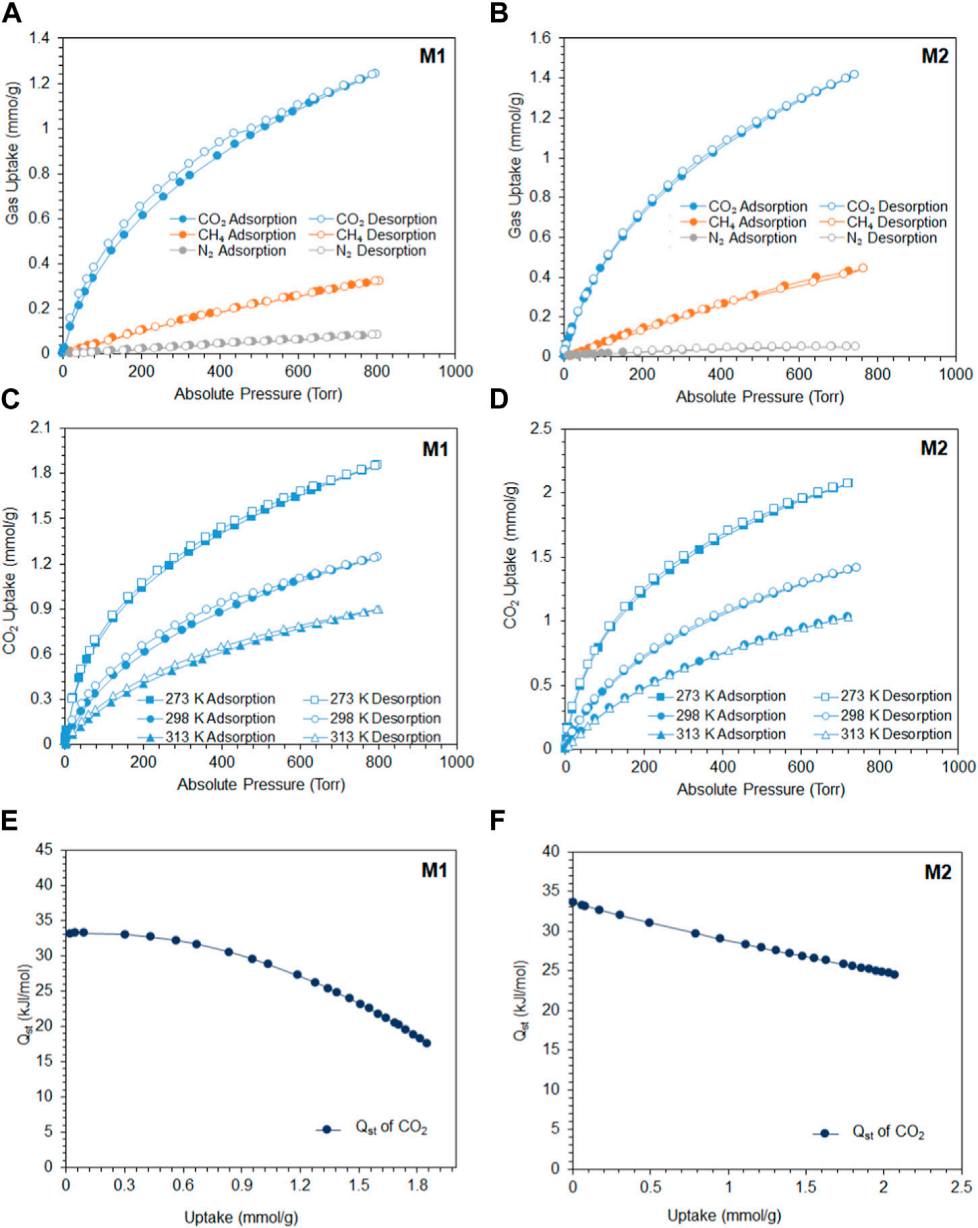
**(A)** Adsorption/desorption of **M1** to CO_2_, CH_4_, and N_2_ at 298 K; **(B)** adsorption/desorption of **M2** to CO_2_, CH_4_, and N_2_ at 298 K; **(C) M1** adsorption/desorption of CO_2_ at 273, 298, and 313 K; **(D) M2** adsorption/desorption of CO_2_ at 273, 298, and 313 K (filled circles refer to adsorption, and unfilled circles refer to desorption); **(E) M1** isosteric heat of adsorption (*Q*_st_) vs CO_2_ uptake (mmol/g); and **(F) M2** isosteric heat of adsorption (*Q*_st_) vs CO_2_ uptake (mmol/g).

**FIGURE 4:**
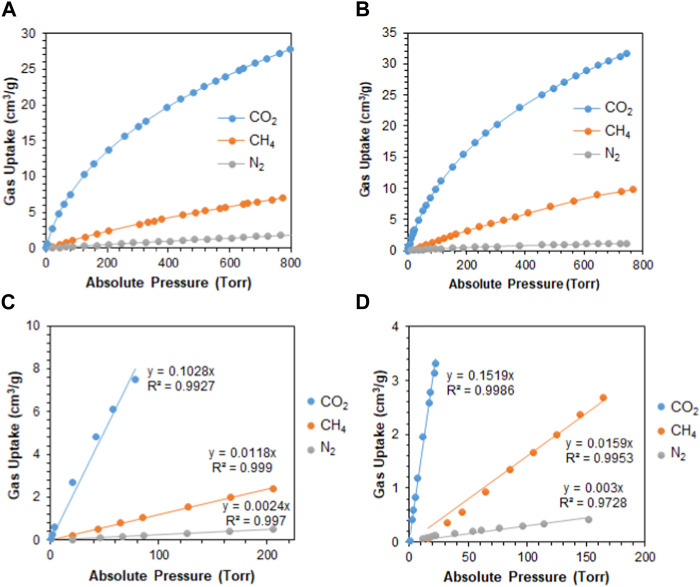
**(A)** Adsorption of CO_2_, CH_4_, and N_2_ by **M1** at 298 K; **(B)** adsorption of CO_2_, CH_4_, and N_2_ by **M2** at 298 K; **(C)** initial slope fitting of **M1** at 298 K; and **(D)** initial slope fitting of **M2** at 298 K.

The tuned pore size distribution, accompanied by the good surface areas, encouraged us to investigate the capabilities of **M1** and **M2** for CO_2_ adsorption compared with CH_4_ and N_2_ for applications in natural gas purification and flue gas treatment. For the polymers to perform well, they should be selective toward CO_2_ and that can be produced by enhancing the microporosity of the polymer. As shown in Figure 2B, **M2** is microporous in nature, with pore size distributions falling in the microporous region less than 20 Å with a high intensity close to the kinetic diameter of CO_2_ (3.3 Å). This is shown by the adsorption capacities observed in Figures 3A–D, where the adsorption capacity at 273 K of CO_2_ is higher in **M2** (2.1 mmol/g) compared to **M1** (1.85 mmol/g). At 298 K, the adsorption capacities behave in a similar manner, where the adsorption capacity of **M2** for CO_2_ is 1.41 mmol/g, for CH_4_ is 0.44 mmol/g, and for N_2_ is 0.050 mmol/g, whereas the adsorption capacity of **M1** for CO_2_ is 1.24 mmol/g, for CH_4_ is 0.32 mmol/g, and for N_2_ is 0.08 mmol/g. Comparing the efficiency between **M1** and **M2**, it is shown that the adsorption capacity of **M2** was higher than **M1**, which is attributed to the microporous nature of the pores and the absence of mesopores in **M2** (Song et al., 2022). The isosteric heat of adsorption (*Q*_st_) of CO_2_ shows the interaction energy between a sorbent and CO_2_ gas. Figures 3E, F show the *Q*_st_ vs uptake of CO_2_. The values of *Q*_st_ decrease with the coverage of the surface of the polymer with CO_2_, indicating that the adsorption process occurred on a heterogeneous surface. The *Q*_st_ values for the adsorption of CO_2_ by **M1** and **M2** were found to be 33.1 kJ/mol and 33.6 kJ/mol, respectively. This indicates that the adsorption process is of physisorption in nature (Khosrowshahi et al., 2022; Ravi et al., 2023). Another feature that an adsorbent should possess is high selectivity. As shown in Figures 4A–D, the selectivity was investigated at 298 K to mimic ambient conditions, which is in agreement with post-combustion treatment conditions. By using the initial slope ratios of Henry’s law constants at 298 K, the selectivity of **M1** for CO_2_/N_2_ is 43 and CO_2_/CH_4_ is 9, whereas the selectivity of **M2** for CO_2_/N_2_ is 51 and CO_2_/CH_4_ is 10. The selectivity of **M2** was higher than that of **M1** even though it has a lower surface area, which could be explained by the microporous nature of the polymer with a similar observation for CO_2_/CH_4_ selectivity. As shown in Table 1, despite having lower surface areas, **M1** and **M2** exhibit superior adsorption capacity and selectivity for CO_2_/CH_4_ and CO_2_/N_2_ compared to reported porous materials with higher surface areas.

**TABLE 1:** Comparison of porous materials with M1 and M2 with respect to surface area, CO_2_ uptake at 273 K and 298 K, CO_2_/N_2_ and CO_2_/CH_4_ selectivity, and *Q*_st_ (CO_2_).

Name	BET (m^2^/g)	CO_2_ (mmol/g) 273 K (298 K)	CO_2_/N_2_ selectivity (298 K)	CO_2_/CH_4_ selectivity (298 K)	*Q*_st_ (CO_2_) kJ/mol	Reference
M1	575	1.85 (1.24)	43	9	33.1	This work
M2	389	2.10 (1.41)	51	10	33.6	
P-C	339	1.32 (0.72)	-	20.7	-	Li et al. (2021)
P-N-ET	1,150	4.0 (2.11)	67.4	36.7	-	
P-C-ET	1,031	2.61 (1.57)	28.7	16.5	-	
SNW-1	821	(2.08)	50	15	35	Gao et al. (2014)
P-1	611	2.02	29	4	38.8	Qiao et al. (2014)
P-2	1,222	3.3	8	3	30.9	
Polymer 1	1,168	2.18 (1.09)	56	-	35.5	Wang et al. (2015)
Polymer 2	1,015	2.08 (1.61)	45	-	27.2	
BIPLP-1Cu/BF_4_	1,580	2.30 (1.20)	16	3	32.2	Arab et al. (2015)
BIPLP-1	380	2.5 (1.75)	64	17	32.3	
YBN-CC	579	2 (1.27)	32.25	5.15	24.7	Sadak (2021)
YBN-DMM	784	2.87 (1.75)	25.78	5.14	26.5	
YBN-DMB	957	2.87 (1.68)	23.19	4.96	28.8	

**FIGURE 5:**
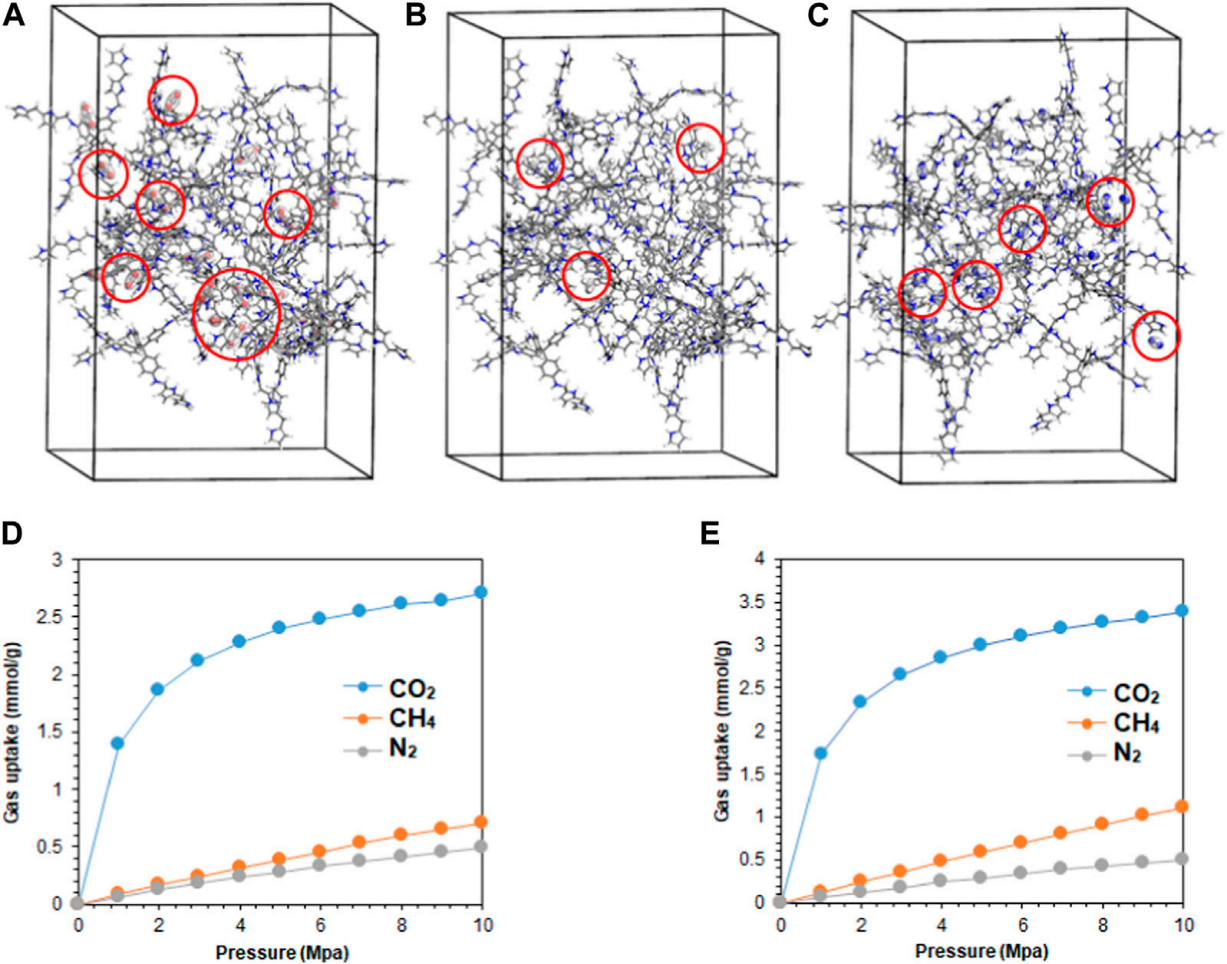
**(A)** CO_2_, **(B)** CH_4_, and **(C)** N_2_ adsorption sites located on the **M2** polymer packed in amorphous cells of dimension 30 × 30 × 40 Å, comprising 20 repeating units. The red spheres represent the adsorbed gas molecules. The corresponding simulated adsorption isotherms for both polymers at 298.15 K are presented in **(D) M1** and **(E) M2**.

**FIGURE 6:**
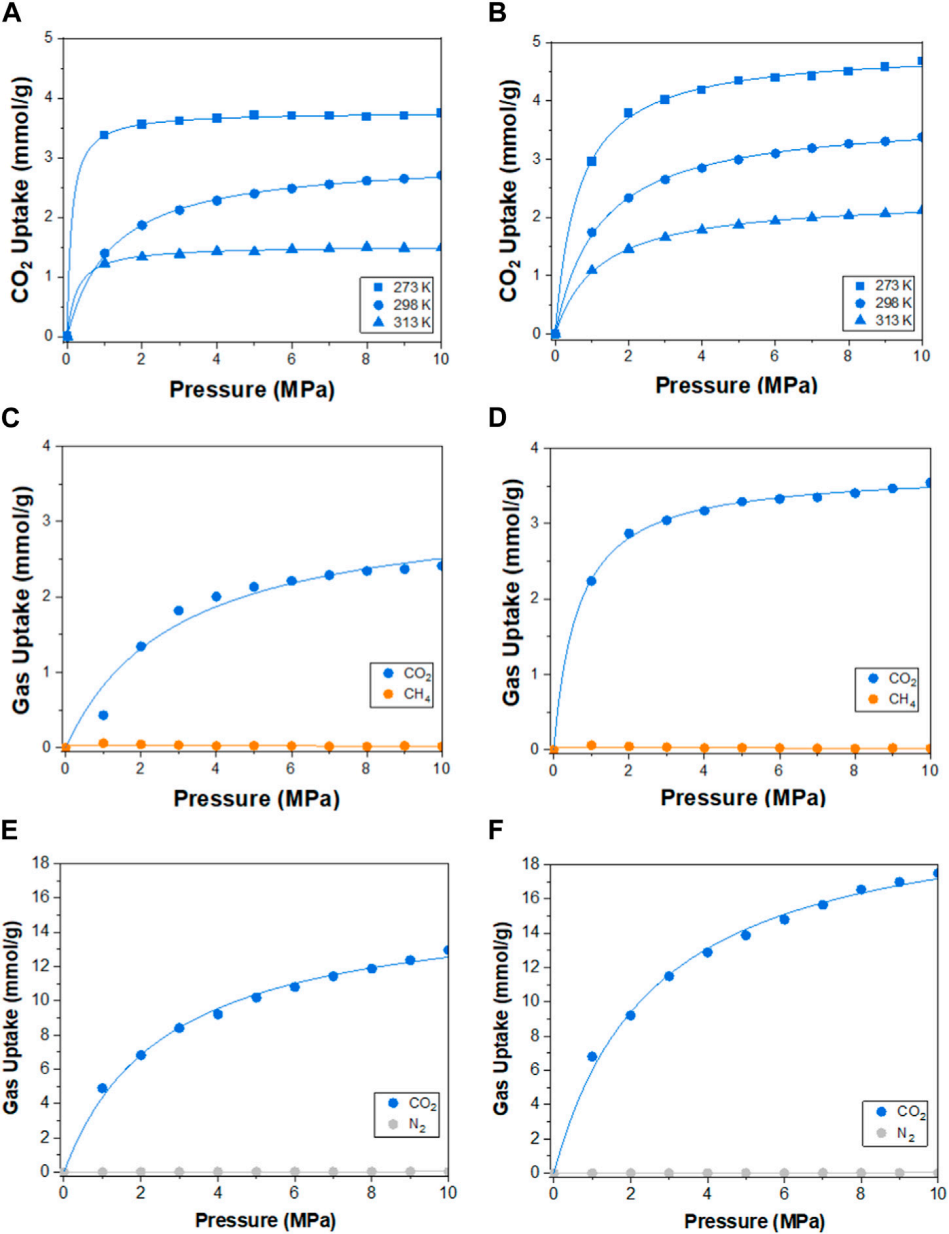
Theoretical adsorption isotherms of CO_2_ at 273.15, 298.15, and 313.15 K on **(A) M1** and **(B) M2**. Adsorption isotherms of CO_2_/CH_4_ in multi-component streams with a molar ratio of 50:50 on **(C) M1** and **(D) M2** at 298.15 K; and CO_2_/N_2_ with a molar ratio of 20:80 on **(E) M1** and **(F) M2** are also presented.

The molecular dynamics of the synthesized polymers were studied to correlate the experimental results with the theoretical calculations (Supplementary Material). The adsorption of single-component gases, CO_2_, CH_4_, and N_2_, on **M1** and **M2** polymers at 298.15 K was simulated in supercells of dimension 30 × 30 × 40 Å, comprising of 20 repeating units of the polymer molecules, as presented in Figures 5A–C. The corresponding simulated adsorption isotherms are shown in Figures 5D, E. Both polymers demonstrated strong van der Waals attraction toward CO_2_ molecules via the pyrrolic and pyridinic nitrogen atoms on **M1** and **M2**, respectively. Moreover, CO_2_ adsorption binding sites were located on both molecules, with fewer sites for CH_4_ and N_2_ gases. However, **M2** demonstrated rapid uptake of CO_2_ below 2 MPa (20 bar), indicating the presence of microporosity within the polymer framework (Rizzuto et al., 2017), and the selectivity of the polymer to CO_2_ gas is consistent with the experimental findings. Using the Langmuir isotherm model (Table 2), the limit adsorption capacities of CO_2_ at 298.15 K on **M1** and **M2** were estimated as 2.99 and 3.74 mmol/g, while for CH_4_ and N_2_, the values were 0.44 and 0.17, and 0.98 and 0.20 mmol/g, respectively. Meanwhile, the corresponding theoretical isosteric heat of adsorption (*Q*_st_) for CO_2_ at 298.15 K was calculated as 43.1 and 43.9 kJ/mol on **M1** and **M2**, respectively. While the theoretical values are slightly higher than the experimental values, which could be ascribed to the overestimation from the general assumptions input into the simulation software (Meng et al., 2018). The order of selectivity of the polymers is in good agreement with the experimental findings and revealed the preferential selectivity of **M2** to CO_2_ gas. The adsorption of CO_2_ on **M1** and **M2** at temperatures of 273.15, 298.15, and 313.15 K was further investigated, and the results are presented in Figures 6A, B. A slight decrease in the adsorption capacity of both polymers was observed with increasing temperature. This suggests that the adsorption of the gas molecules is strictly dependent on the van der Waals force of attraction between them and the active sites on the polymers, which tend to weaken with the increase in temperature due to the increase in the inherent kinetic energy of the gas molecules. Thus, **M2** experiences less decline in the adsorption capacity, suggesting its greater adsorption preference for CO_2_ gas.

**TABLE 2:** Langmuir fitting parameters for the adsorption of CO_2_, CH_4_, and N_2_ on M1 and M2.

Temperature (K)	Polymer	Gas	a (mmol/g)	b (Mpa^−1^)	R^2^
273.15	M1	CO_2_	3.77	0.12	0.9998
	M2		4.90	0.64	0.9995
298.15	M1	CO_2_	2.99	0.12	0.9994
	M2	CO_2_	3.74	0.12	0.9995
	M1	CH_4_	0.44	0.52	0.9999
	M2	CH_4_	0.98	0.78	0.9999
	M1	N_2_	0.17	0.27	0.9999
	M2	N_2_	0.20	0.30	0.9999
313.15	M1	CO_2_	1.52	0.26	0.9998
	M2		2.34	0.19	0.9994

Meanwhile, the selectivity of the polymers toward CO_2_ in multi-component gas streams, comprising CO_2_ and CH_4_ at a molar ratio of 50:50 and CO_2_ and N_2_ gases at a molar ratio of 20:80, was further explored theoretically, as shown in Figures 6C–F. The presence of equimolar volumes of CH_4_ and the abundance of N_2_ gases did not impede the selectivity of the polymers to CO_2_ gas, as the presence of quadrupole C=O bonds favors the van der Waals attraction to the polymers. On the other hand, CH_4_ and N_2_ molecules experienced a drastic decrease in adsorption affinity by the polymers due to the strong competition by CO_2_ molecules, resulting in fewer molecular interactions, as shown in Figures 6C–F. In all cases, the **M2** polymer rapidly adsorbs CO_2_ molecules in the presence of competing gas molecules, affirming its selectivity and aligning well with the experimental findings.

**FIGURE 7:**
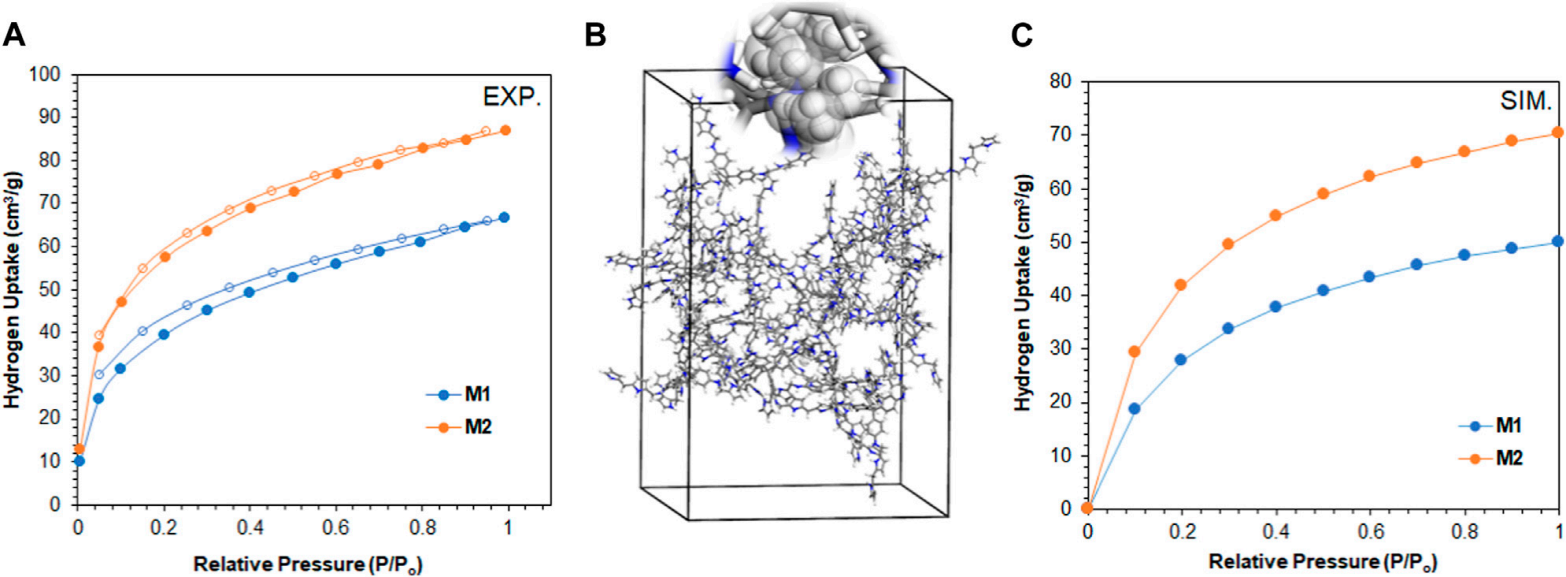
**(A)** H_2_ adsorption/desorption isotherm of **M1** and **M2** at 77 K (filled circles refer to adsorption, and unfilled circles refer to desorption). **(B)** Supercell of dimension 30 × 30 × 40 Å, comprising 20 repeating units of **M2** for the simulation of H_2_ storage capacity and **(C)** theoretical adsorption isotherms of H_2_ at 77 K on **M1** and **M2**.

In our endeavor to tap into the world of clean energy and seek alternatives to overcome the pollution of petroleum products, we assessed our polymers for their capability to store hydrogen gas. The results in Figure 7A revealed the adsorption capacity of **M1** and **M2** toward H_2_ at 77 K and 1 atm to be 66 cm^3^/g (0.6 wt%) and 87 cm^3^/g (0.8 wt%), respectively. Interestingly, the absence of mesopores in **M2** reflected the higher adsorption capacity toward H_2_ compared to **M1**, which is consistent with Rong et al. (2021). The storage capacity is higher and comparable to that of porous polymers with similar or higher surface areas, as shown in Table 3. Finally, we simulate the potential of hydrogen storage on the polymer molecules by constructing supercells and conducting the grand canonical Monte Carlo simulation, as shown in Figures 7B, C. The theoretical H_2_ uptake capacities of **M1** and **M2** at 77 K were calculated to be 61.7 and 83.2 cm^3^/g, respectively. These values are in good agreement with the experimental adsorption capacities of both polymers and corroborate the microporosity of **M2**, making it a potential material for H_2_ storage.

**TABLE 3:** Comparison of porous materials to M1 and M2 for hydrogen storage.

**Material**	**BET (m** ^**2**^ **/g)**	**H**_**2**_ **wt%** ^a^	**Reference**
M1	575	0.6	This work
M2	389	0.8	
Poly (styrene-co-divinylbenzene)	1,060	0.8	Germain et al. (2009)
PIM-1	760	1.04	McKeown et al. (2006)
Fluoropolymers with intrinsic microporosity	666	0.8	Makhseed et al. (2008)
SCMP1	505	0.77	Cheng et al. (2012)
PCZN-10	391	0.75	Liao et al. (2017)
Bipyridinium array-type porous polymer	-	0.71	Yao et al. (2009)
ZSM-5	431	0.7	Dybtsev et al. (2004)

^a^ Data based on 77 K and 1 bar.

## 4 Conclusion

In this study, we report the synthesis of 3D porous polymers with tuned porosity. The choice of reaction conditions and monomers leads to polymers with microporous and meso-/microporous structures. The produced polymers were found to be thermally stable up to temperatures of 400°C. Analysis revealed the porous nature of polymers with a BET surface area of 575 m^2^/g for **M1** and 389 m^2^/g for **M2**. The **M1** polymer showed defined micropores of 7 Å and mesopores of 33 Å, whereas **M2** exhibited micropores with a pore size distribution of <20 Å. The study also revealed the effect of microporosity on adsorption ability and selectivity. The results revealed superior performance of **M2** in the absence of mesoporosity. The adsorption capacities at 273 K of CO_2_ are higher in **M2** (2.1 mmol/g) compared to **M1** (1.85 mmol/g). In addition, at 298 K, the adsorption capacity of **M2** for CO_2_ was 1.41 mmol/g, for CH_4_ was 0.44 mmol/g, and for N_2_ was 0.050 mmol/g, while the adsorption capacity of **M1** for CO_2_ was 1.24 mmol/g, for CH_4_ was 0.32 mmol/g, and for N_2_ was 0.08 mmol/g. Furthermore, the absence of mesoporosity in M2 was evidenced by its superior performance in hydrogen storage. The molecular dynamics simulation confirmed the superior performance of **M2** and coincided with the experimental values to prove the efficiency and capability of porous polymers to be a potential adsorbent for selective removal of CO_2_ and H_2_ storage.

## Data Availability

The original contributions presented in the study are included in the article/Supplementary Material; further inquiries can be directed to the corresponding author.
