# Supplementary material for: 3D porous polymers for selective removal of CO2 and H2 storage: experimental and computational studies
Source: Front Chem. 2023 Sep 7;11:1265324. doi: 10.3389/fchem.2023.1265324 (PMC10513180; doi:10.3389/fchem.2023.1265324)
Supplement: Supplementary file 1 [file DataSheet1.docx]

**Supplementary Information**

**3D Porous Polymers for Selective Removal of CO_2_ and H_2_ Storage: Experimental and Computational Studies**

Muath S. Al-Bukhari^a^, Ismail Abdulazeez^b^, Mahmoud Abdelnaby^c^, Isam H. Aljundi^b,d^ , Othman Charles S. Al Hamouz*^a,c^

*^a^Chemistry Department, King Fahd University of Petroleum & Minerals, Dhahran 31261, Saudi Arabia*

*^b^Interdisciplinary Research Center for Membranes and Water Security, King Fahd University of Petroleum & Minerals, Dhahran 31261, Saudi Arabia*

*^c^Interdisciplinary Research Center for Hydrogen and Energy Storage, King Fahd University of Petroleum and Minerals, Dhahran 31261, Saudi Arabia*

*^d^Chemical Engineering Department, King Fahd University of Petroleum and Minerals, Dhahran 31261, Saudi Arabia*

*To whom correspondence should be addressed: othmanc@kfupm.edu.sa

***MD simulation***

Insights on the porous natures of **M1** and **M2** and the gas separation features were investigated using classical molecular dynamics simulations. Amorphous simulation cells of dimensions 30 × 30 × 30 Å comprising of 20 repeating units of the porous materials were constructed and geometrically relaxed using the Forcite module. The cells were subjected to dynamics simulations on the NPT and the NVT ensembles each for 1000 ps, at a timestep of 0.001 ps, and temperatures of 273.15, 298.15 and 313.15 K. The Nose-Hoover thermostat and the Berendsen barostat were used for the control of temperature and the pressure, respectively, while the Ewald summation method was used to treat the long-range Coulombic interactions. Lastly, the Lennard-Jones attractive and repulsive interactions were estimated within the cut-off range of 18.5 Å. The geometrically-relaxed cells are presented in figure 1S. The blue-grey isosurface plots at probe radius of 1.20 Å depicts the free accessible volumes on the polymer materials, and corresponds to an estimated free fractional volume (FFV) of 0.33 and 0.35 on **M1** and **M2**, at 273.15, 298.15 and 313.15 K, respectively. Meanwhile, the Connolly surface area on both materials were estimated at 16800, 16850 and 16880 Å^2^ on **M1**, and 17200, 17220 and 17240 Å^2^ on **M2**, at 273.15, 298.15 and 313.15 K, respectively. These results suggest the porosity of the membranes and revealed the presence of larger pore volumes and higher surface area on **M2**. It also demonstrates the compactness, the thermal stability, and the gas permeability of both membranes, making them suitable for gas separation and purification.


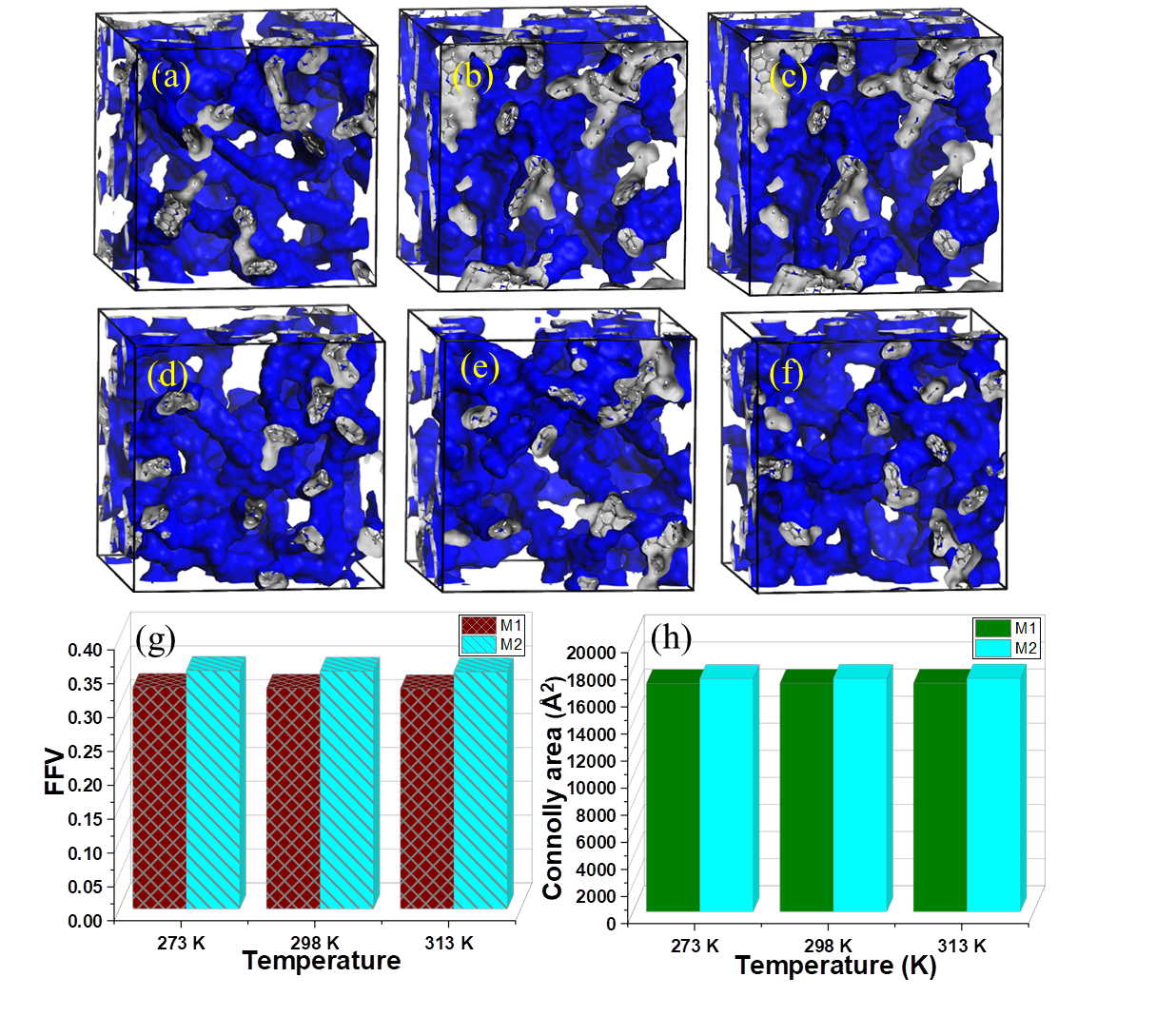


**Fig. 1S**. Geometrically optimized amorphous cells comprising of 20 repeating units of **M1** (a-c) and **M2** (d-f) at 273.15, 298.15 and 313.15 K, respectively. The estimated FFV of the polymers at the specified temperatures are presented in (g), while the Connolly surface area are shown in (h).

| **Table 3**  Langmuir fitting parameters for the adsorption of CO_2_, CH_4_ and N_2_ on **M1** and **M2.** | | | | | |
| --- | --- | --- | --- | --- | --- |
| **Temperature (K)** | **Polymer** | **Gas** | **a (mmol/g)** | **b (Mpa^-1^)** | **R^2^** |
| 273.15 | M1  M2 | CO_2_ | 3.77  4.90 | 0.12  0.64 | 0.9998  0.9995 |
| 298.15 | M1  M2  M1  M2  M1  M2 | CO_2_  CO_2_  CH_4_  CH_4_  N_2_  N_2_ | 2.99  3.74  0.44  0.98  0.17  0.20 | 0.12  0.12  0.52  0.78  0.27  0.30 | 0.9994  0.9995  0.9999  0.9999  0.9999  0.9999 |
| 313.15 | M1  M2 | CO_2_ | 1.52  2.34 | 0.26  0.19 | 0.9998  0.9994 |
